# On the occurrence of *Astrospartus mediterraneus* (Echinodermata, Gorgonocephalidae) in the Northwestern Iberian Peninsula

**DOI:** 10.7717/peerj.21268

**Published:** 2026-05-12

**Authors:** Bruno Almón, Jacinto Pérez-Dieste

**Affiliations:** 1 Grupo de Estudo do Medio Mariño (GEMM), Ribeira, A Coruña, Spain; 2 Centro Oceanográfico de Vigo, Instituto Español de Oceanografía (IEO, CSIC), Vigo, Pontevedra, Spain

**Keywords:** Distribution, Taxonomy, Marine biology, Climatic change, Larval dispersion, Northward expansion

## Abstract

The first record of a living specimen of *Astrospartus mediterraneus* (Risso, 1826) in Galician waters is presented, representing a significant extension of the known northern limit for this charismatic, yet little-known, species of ophiuroid. Traditionally restricted to the western Mediterranean Sea and adjacent Atlantic, *A. mediterraneus* is characterized by its cryptic habits and association with deep benthic communities dominated by gorgonians and sponges. The specimen observed in Galicia was found in a shallow subtidal habitat, at the edge of the mesophotic zone considered compatible with its ecological requirements. This finding raises new hypotheses regarding population connectivity between the Mediterranean Basin and Atlantic regions, as well as the role of ocean currents and climate change in the redistribution of benthic species. The biogeographic implications of this record are discussed, and the need to intensify monitoring efforts for deep-sea species in the Iberian Atlantic is proposed.

## Introduction

The basket star *Astrospartus mediterraneus* (Risso, 1826) is an emblematic euryalid species and the only representative of the family Gorgonocephalidae found along the Spanish littoral (Ocaña & Pérez-Ruzafa, 2004; Biel-Cabanelas et al., 2023). As a member of the family Gorgonocephalidae, it is distinguished by its highly branched arms, which are adapted for suspension feeding in deep benthic environments. This species has traditionally been regarded as endemic to the Mediterranean Sea and the northeastern Atlantic, with confirmed occurrences in regions such as the Ligurian Sea, coast of Corsica, and southern Iberian Peninsula (Ocaña et al., 2022; Canessa, Trainito & Bavestrello, 2024). Although several isolated records from the northern Portuguese coast have been reported, these are infrequent and typically involve specimens captured by trawl fishing gear or dead individuals of unknown origin (GBIF.org, 2025).

Despite being considered an emblematic species in the Mediterranean Sea, it has not received much attention from the scientific community until recently, when several studies have been published in response to high aggregations, first detected due to their interference with fishing gears, hampering regular fishing activities (Biel-Cabanelas et al., 2023; Canessa et al., 2023; Canessa, Trainito & Bavestrello, 2024). Thus, knowledge regarding its ecological traits and dispersion mechanisms is still scarce, a circumstance that is extensible to most species in the Euryalida order (Calero & Ramil, 2023).

General knowledge on the biology of *A. mediterraneus* reveals cryptic and nocturnal behavior, with a strong association with sessile organisms such as gorgonians and sponges, which it utilizes as support to extend its arms for feeding. Recent studies have documented larval recruitment processes in habitats dominated by *Paramuricea clavata* (Risso, 1827) and *Eunicella verrucosa* (Pallas, 1766), suggesting an ecological dependence on these communities for its establishment and persistence (Canessa, Trainito & Bavestrello, 2024). Although previous records in the Eastern Atlantic suggested a northward extension of the species distribution along the Portuguese coasts, information about these specimens is often limited, and is usually based on dead individuals obtained as bycatch during fishing operations (GBIF.org, 2025).

The Galician region, located in the northwest corner of the Iberian Peninsula, represents the northern boundary of the Canary upwelling system, at the transition between the subtropical and subpolar regimes of the North Atlantic, which makes it of particular interest for the detection of marine ecosystem changes (Bañón et al., 2024). In recent years, reports on species expanding their distribution northwards have been increasing, with examples belonging to different phyla reaching this region (Cuesta et al., 2016; Vale et al., 2021; Almón et al., 2022; Bañón et al., 2002; Bañón et al., 2024; Rius et al., 2024).

In this context, the discovery of a living specimen of *A. mediterraneus* in Galician waters is a biogeographically significant event, representing an expansion of the known northern limit for the species. This new record prompts inquiries into population connectivity between the Mediterranean and the Atlantic, as well as the potential impacts of climate change and oceanographic alterations on the redistribution of benthic species.

This contribution documents this unprecedented finding in Galicia, providing morphological, ecological, and geographical data on the observed specimen. Furthermore, the implications of this record are discussed in the context of the marine biogeography of the Iberian Atlantic, and the need to intensify monitoring efforts in mesophotic and deep habitats to detect potentially established populations is proposed.

## Materials & Methods

**Figure 1:**
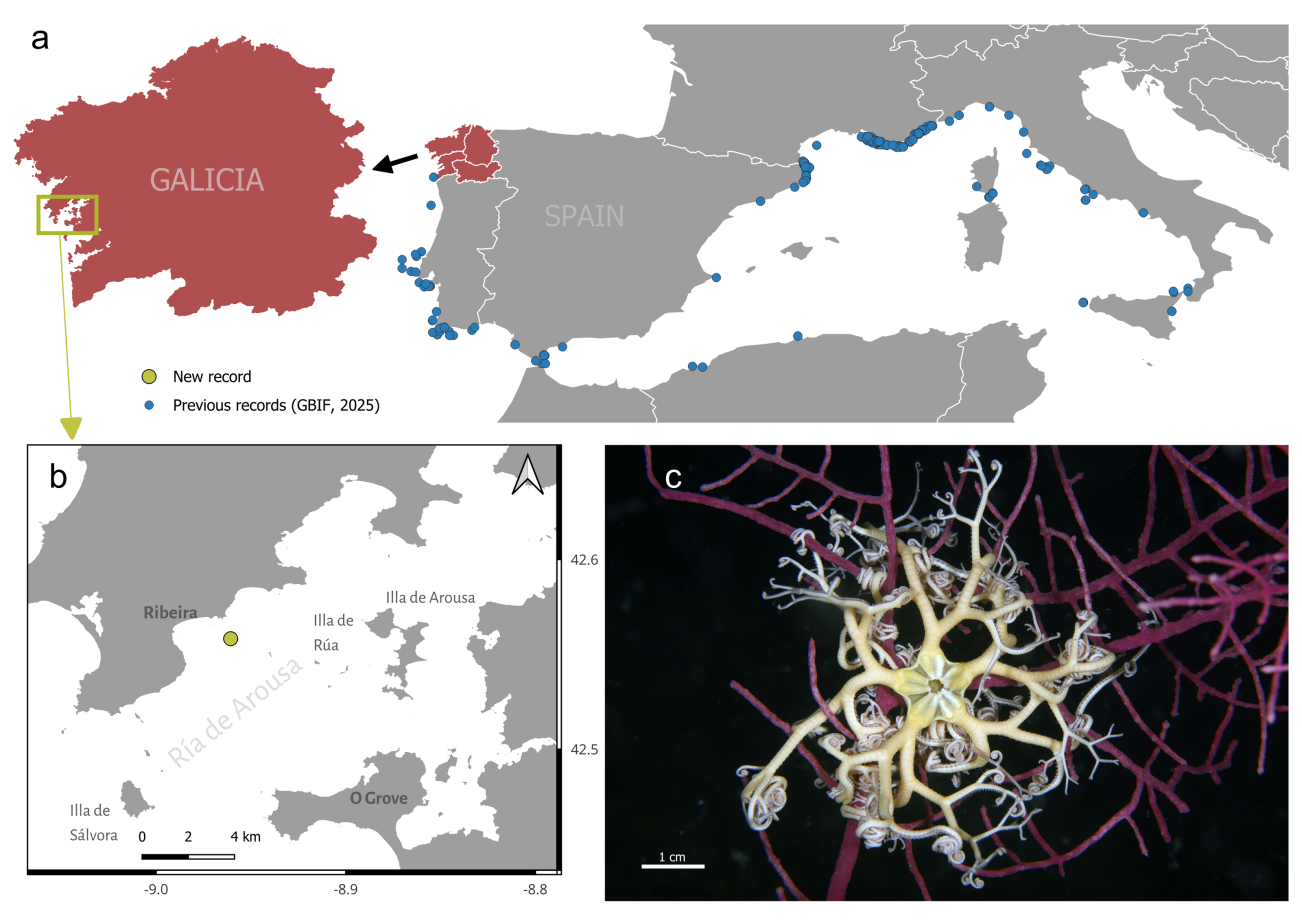
Distribution of *Astrospartus mediterraneus* (Risso, 1826) in Mediterranean and Atlantic waters. (A) Blue dots indicate published and unpublished records obtained from GBIF. The enlarged red area indicates the study region. (B) Detail of the study area indicating the location of the sampling site where the live specimen was found. (C) Underwater image of the living specimen among the branches of the gorgonian *Leptogorgia sarmentosa*. Scale bar: *c* = 1 cm.

Between 2022 and 2025, a series of benthic surveys using SCUBA diving to evaluate changes in marine fauna biodiversity along the Galician coasts were conducted by the research team of the NGO “Grupo de Estudo do Medio Mariño” (GEMM). In June 2025, during one of the transects near the dive site known as “A Tartaruga,” a living specimen of an ophiuroid assignable to the Gorgonocephalidae family was discovered (Fig. 1). The specimen was photographed in its natural habitat and subsequently preserved in 70° alcohol for further examination in the laboratory. It was then examined and photographed under a Leica M165C stereomicroscope with Leica DMC4500 digital camera. Following morphological study, the specimen was assigned to the species *Astrospartus mediterraneus*. Additional measurements were taken on the disc diameter, measured from center to the interradial edge of the disc.

Molecular taxonomic analysis was conducted by sequencing the mitochondrial cytochrome oxidase subunit I (COI) and mitochondrial 16S rRNA (16S) genes of the putative *A. mediterraneus* specimen. DNA was extracted from 25 mg tissue following the spin column protocol of the EZNA Tissue DNA Extraction Kit (Omega Bio-Tek). The standard 5′ barcoding region of the *COI* gene was amplified by polymerase chain reaction (PCR) using the primer pair LoboF1 and LoboR1 (Lobo et al., 2013), and the 16S region using the primers 16Sar-5′ and 16Sbr-3′ (Ivanova et al., 2007). The following reaction conditions were applied: initial denaturation at 94 °C for 5 min followed by 35 cycles of 94 °C for 35 s, annealing at 48 °C for 35 s and 72 °C for 90 s, with a final extension at 72 °C for 7 min. PCR was carried out using Canvax Horse Power Taq DNA polymerase (Canvax Biotech, Spain); mixtures contained a final volume of 20 µl and included 10 µl of 2 × Taq DNA polymerase Master Mix, 2 µl of primer cocktail and between 50 and 100 ng of template DNA. PCR products were purified by treatment with ExoSAP-IT, subjected to sequencing reactions using the BigDye v3.1 cycle sequencing kit, and sequenced in both forward and reverse directions using the same primers as in PCR reactions. The sequencing reactions were migrated in a SeqStudio Genetic Analizer (Applied Biosystems, Foster City, CA, USA) at the “Centro de Apoyo Científico y Tecnológico a la Investigación” (CACTI) facilities of Vigo University. Trace files quality was verified using MEGA v11 (Tamura, Stecher & Kumar, 2021), and sequences were queried using the National Center for Biotechnology Information (NCBI) BLASTn tool (Morgulis et al., 2008) for taxonomic comparison. The newly generated sequences for the COI and 16S genes have been deposited in GenBank under Accession codes PZ161872 and PZ161128, respectively, and the voucher specimen in the invertebrate collection of the “Museo de Historia Natural Luis Iglesias”, in Santiago de Compostela, under reference code MHN USC-25256.

## Results

**Table:** 

**Gorgonocephalidae Ljungman, 1867**
*Astrospartus* Döderlein, 1911
*Astrospartus mediterraneus* (Risso, 1826) Figs. 1 and 2
*Astrospartus arborescens* (L. Agassiz, 1839)
*Euryale arborescens* L. Agassiz, 1839
*Euryale mediterraneus* Risso, 1826
*Gorgonocephalus arborescens* (L. Agassiz, 1839)
*Gorgonocephalus verrucosus* Grube, 1840

Material examined: one living specimen (MHN USC-25256), disc radius = 11 mm, “A Tartaruga” shelf, 42°32′34.9″N, 8°57′19.1″W, captured at 33 m depth on June 18, 2025, on a rocky bottom covered with branched octocorals.

**Figure 2:**
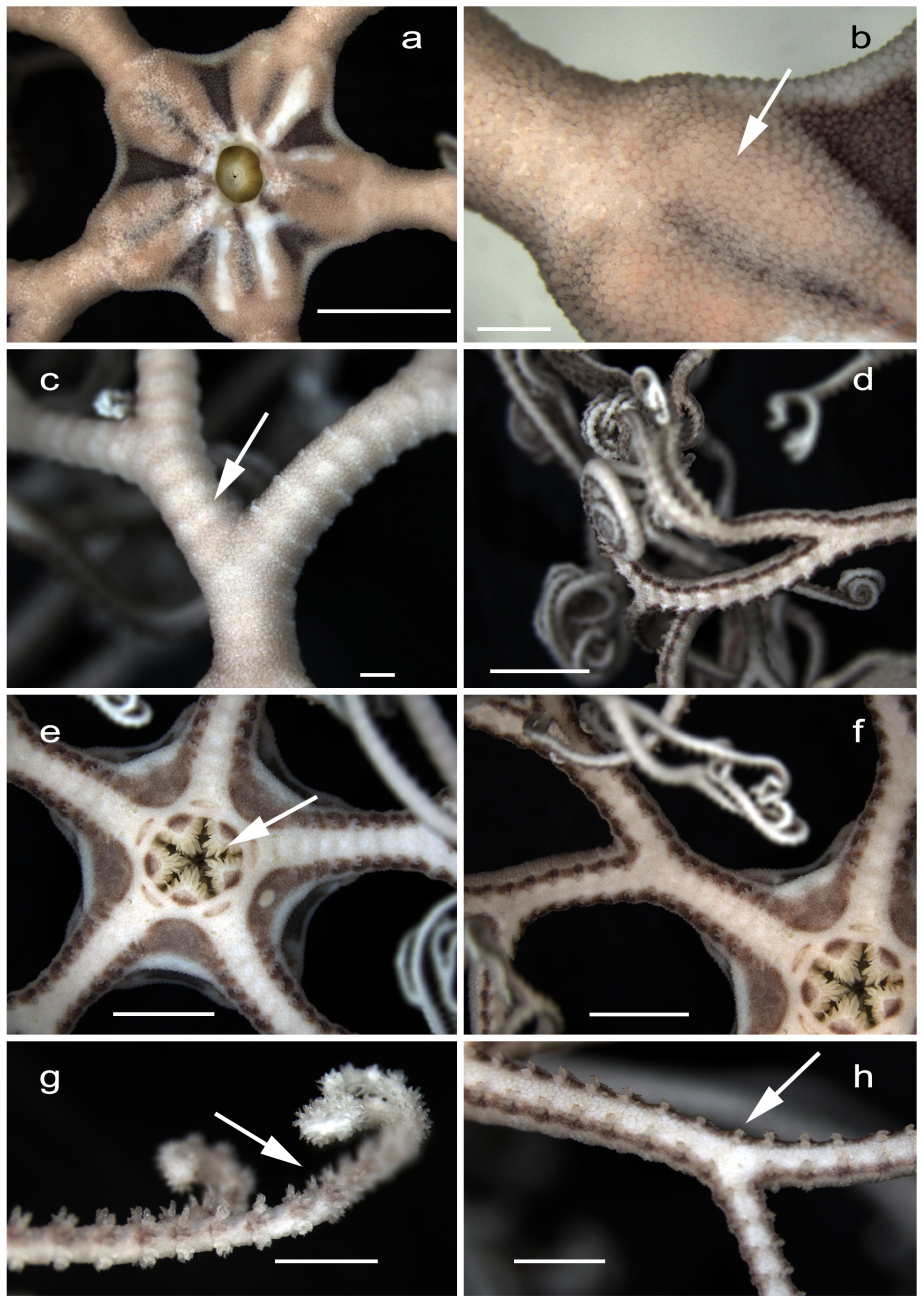
Morphological features of *Astrospartus mediterraneus*. (A) Dorsal surface of the disc. (B) Dorsal view of disc periphery. (C) Dorsal view of first branch of an arm. (D) Distal portion of arms. (E) Ventral disc. (F) Ventral view of arm base. (G) Variations in arm spines along the arm. (H) Ventral view of secondary branch of an arm. Scale bars: a, d–f = 5 mm; b–c, g–h = 1 mm.

Description: Disc pentagonal, covered with small granulations that extend along the branched arms (Figs. 2A, 2B). Dorsal surface with five pairs of radial ribs tapering towards the center, where they converge; areas between ribs hollowed and darker. Oral surface with very pronounced closed ambulacral grooves; dental plates entire, bearing multiple isometric spine-shaped teeth arranged in irregular columns (Fig. 2E). Five primary arms that bifurcate repeatedly near the base and along their length, with vertebrae that articulate by closely spaced ossicles (Figs. 2C, 2D). Arms with strong conical spines ventrally, that form bands or rows as they branch out (Figs. 2D, 2F, 2H), giving it a rough appearance to the touch and becoming small hook-like projections distally (Fig. 2G). Overall coloration yellowish, more intense at the oral radial plates, fading in the arms toward the tips.

Habitat: The specimen was observed at 33 m depth, fully extended, with arms twisted around gorgonian branches. The specimen was among the branches of the gorgonian *Leptogorgia sarmentosa* (Esper, 1791), a common species in the area (Fig. 1) frequently forming dense aggregations on rocky bottoms.

Distribution: Found in the western Mediterranean and adjacent Atlantic waters along the coasts of Spain, Portugal, Senegal, Algeria, and Morocco. It lives at depths between 30 and 800 m, usually on branches of gorgonians of the genera *Paramuricea* Kölliker, 1865, *Eunicella* Verrill, 1869, and *Leptogorgia* Milne Edwards, 1857, or on large sponges such as *Aplysina cavernicola* (Vacelet, 1959), *Sarcotragus foetidus* Schmidt, 1862, *Spongia lamella* (Schulze, 1879), or *Axinella polypoides* Schmidt, 1862.

Remarks: This species has mainly been recorded in association with *Paramuricea clavata* in the Mediterranean. Although species of the genus *Paramuricea* have also been recorded in Galicia, its abundance is much lower, being replaced in importance by *Leptogorgia sarmentosa* in shallower areas and *Eunicella verrucosa* (Pallas, 1766) at greater depths. Some differences observed, such as coloration, could provide information about ecological differences or simply reflect the individual’s stage of maturity. In the literature, this coloration is described as uniform, pinkish brown, even in small individuals (Canessa, Trainito & Bavestrello, 2024), while the specimen described here was generally lighter in color, more yellowish in the body, fading to a bright white towards the tips of the arms.

Molecular Analysis: The molecular analysis revealed that the newly generated sequences exhibited low genetic variability compared with the available sequences from Mediterranean specimens. Specifically, the COI gene sequences demonstrated a high degree of similarity, with maximum identity values ranging from 99.5% to 99.7%, while the 16S gene sequences exhibited even greater conservation, with similarity values between 99.8% and 100%. Overall, molecular evidence suggests a strong genetic homogeneity among *A. mediterraneus* populations across regions, supports the morphological identification, and provides an additional genetic reference for *A. mediterraneus* in public databases for two different markers.

## Discussion

The record of *Astrospartus mediterraneus* in Galician waters represents a significant northward extension of the known range for this species, whose stable populations had previously been documented mainly in the western Mediterranean and the southern sectors of the northeastern Atlantic (Biel-Cabanelas et al., 2023; Canessa, Trainito & Bavestrello, 2024). Moreover, considering the sparse records along the Atlantic coasts of Portugal, this finding raises important questions about the mechanisms of larval dispersal and the connectivity between Mediterranean and Atlantic populations.

The presence of *A. mediterraneus* in Galicia could be explained by larval transport processes facilitated by ocean currents such as the northwest Iberian coastal upwelling system, which may act as vectors for dispersal to more northern latitudes (Nolasco et al., 2013). Although developmental data on *A. mediterraneus* remains limited, the presence of large yolky oocytes suggests a lecithotrophic or direct developmental mode (Hendler, 1991). This strategy implies a relatively short planktonic larval duration, ranging from days to weeks, which would favor local recruitment as a primary driver of population structure (Canessa, Trainito & Bavestrello, 2024). Based on this scenario, the presence of a juvenile specimen in Galicia would imply the existence of stable populations that have not yet been recorded along the Portuguese coast. However, population dynamics of *A. mediterraneus* in the Mediterranean appear to show some variability closely linked to environmental fluctuations. Factors such as increased food availability, rising temperatures, and phytoplankton blooms could potentially enhance reproductive output and larval survival (Biel-Cabanelas et al., 2023). The limited genetic variation observed between Galician and Mediterranean specimens suggests greater connectivity between the populations than expected. However, given the scarcity of genetic data available on the species, further studies are needed to gain a deeper understanding of its population dynamics and biogeographical patterns.

Furthermore, the success of colonization depends not only on dispersal potential but also on the availability of suitable settlement conditions in new habitats. The observation of a juvenile specimen at this novel latitude could indeed represent the beginning of a range expansion driven by changing oceanographic conditions, particularly in terms of increasing average temperatures (Gómez-Gesteira et al., 2011; Vale et al., 2021; Biguino et al., 2023). Temperature data from the collection site show increases of approximately 0.78 °C in oceanic and 0.32 °C in coastal waters over the last four decades (Relvas, Luis & Santos, 2009), with current mean temperatures ranging from 14.6 °C to 19.0 °C (Ribeiro et al., 2025). Although direct physiological studies remain absent, these values fall within the known thermal tolerance range for Mediterranean populations (10.1 to 24.3 °C) (Biel-Cabanelas et al., 2023), indirectly supporting the feasibility of establishment in Galician waters. Nevertheless, the alternative hypothesis of a vagrant individual cannot be excluded due to the absence of additional records, leaving open the possibility of an isolated occurrence rather than a stable population.

The lack of prior records despite extensive scientific and commercial monitoring in Galicia suggests that this finding is not simply an overlooked presence but a novel event. Similar range expansions facilitated by warming waters have recently been documented in other echinoderms (Vasco-Rodrigues, 2016; Schäfer, 2023). To clarify the status of this observation, continued monitoring is essential to detect further individuals, assess reproductive activity, and evaluate the potential for long-term establishment, thereby distinguishing between vagrancy and genuine range shift.

The habitat preferences of *A. mediterraneus* are closely tied to the presence of structuring gorgonians, particularly *Paramuricea clavata* and *Eunicella verrucosa* in the Mediterranean, which typically inhabit low-light environments (Coelho et al., 2023). In the Atlantic, *P. clavata* is largely replaced by its sister species *P. grayi*, previously thought to extend to Galicia. However, recent evidence indicates greater diversity within the genus *Paramuricea*, suggesting that the species present in Galician waters may be a different species yet to be described (Ocaña et al., 2022). Moreover, the frequency of occurrence of this species in Galicia is lower compared to the Mediterranean. Dominant gorgonians in Galicia, such as *Leptogorgia sarmentosa* and *Eunicella verrucosa* (Altuna, Sinniger & Aldrey, 2010; Pérez & Almón, 2021), have also been reported as suitable substrata for *A. mediterraneus* (Canessa et al., 2023).

Previous studies have shown that species distribution is mainly determined by habitat requirements, primarily related to the existence of rocky substrates with gorgonians located between 50 and 80 m depth and preferentially occurring on sloping areas (Boavida et al., 2016). The presence of a specimen at a relatively shallow depth suggests that similar environmental conditions, particularly in terms of temperature and light intensity influenced by water turbidity, may be present. The high productivity of Galician waters, derived from the upwelling phenomenon (Casabella, Lorenzo & Taboada, 2014), has a variable effect on turbidity and, therefore, on the extension of the photic zone, which occupies a narrower range compared with that in the Mediterranean (Varela-Rodríguez et al., 1987; Castellan et al., 2022), which might contribute to create suitable conditions for the species.

This new record underscores the importance of monitoring programs in mesophotic and deep habitats, which have traditionally been less explored, for detecting shifts in benthic species distributions. The potential range expansion of *A. mediterraneus* may have ecological implications in terms of interactions with local species and the structure of benthic communities. Although in its early stages, the establishment of the species in the area could lead to proliferations similar to those reported in the Mediterranean (Biel-Cabanelas et al., 2023; Canessa et al., 2023), with implications for ecosystem stability and associated human activities in the future.

Overall, this finding enriches our understanding of echinoderm biogeography in the Iberian Atlantic and highlights the need for integrative studies combining population genetics, physical oceanography, and benthic ecology to elucidate distribution patterns and connectivity.

This new record suggests the existence of more efficient larval dispersal mechanisms than previously considered, while highlighting the possible influence of oceanographic and ecological factors in favoring the establishment of the species at more northern latitudes. The presence of suitable habitats in Galicia, such as shallow mesophotic bottoms dominated by gorgonians, may facilitate the colonization of this region by the species, although the possibility of an isolated event remains. Additionally, this record provides valuable information for understanding the biogeographical patterns of echinoderms in the context of climate change and the dynamics of deep benthic communities.

## Additional Information and Declarations

### Data Availability

The following information was supplied regarding data availability: The voucher specimen is available at the invertebrate collection of the Museo de Historia Natural Luis Iglesias: reference number MHN USC-25256. - The museum collection is not currently available online. To access the voucher specimen, please contact the curator Juan Manuel Cutrín at museohn@usc.es The raw sequence data are available at NCBI BioProject: PZ161872, PZ161128.
